# Complexes of Cobalt(II) Iodide with Pyridine and Redox Active 1,2-Bis(arylimino)acenaphthene: Synthesis, Structure, Electrochemical, and Single Ion Magnet Properties

**DOI:** 10.3390/molecules25092054

**Published:** 2020-04-28

**Authors:** Dmitriy S. Yambulatov, Stanislav A. Nikolaevskii, Mikhail A. Kiskin, Tatiana V. Magdesieva, Oleg A. Levitskiy, Denis V. Korchagin, Nikolay N. Efimov, Pavel N. Vasil’ev, Alexander S. Goloveshkin, Alexey A. Sidorov, Igor L. Eremenko

**Affiliations:** 1 N. S. Kurnakov Institute of General and Inorganic Chemistry, Russian Academy of Sciences, 31 Leninsky prosp., 119991 Moscow, Russian; mkiskin@igic.ras.ru (M.A.K.); nnefimov@yandex.ru (N.N.E.); anubisvas@gmail.com (P.N.V.); sidorov@igic.ras.ru (A.A.S.); ilerem@igic.ras.ru (I.L.E.); 2 Lomonosov Moscow State University, Deptartment of Chemistry, Leninskie Gory 1/3, 119991 Moscow, Russia; tvm@org.chem.msu.ru (T.V.M.); levicoleg@mail.ru (O.A.L.); 3 Institute of Problems of Chemical Physics, Russian Academy of Sciences, Chernogolovka, 142432 Moscow Region, Russia; korden@icp.ac.ru; 4 Nesmeyanov Institute of Organoelement Compounds, 119991 Moscow, Russia; golov-1@mail.ru

**Keywords:** cobalt(II) complexes, redox active ligand, cyclic voltammetry, single ion magnet, dpp-bian, acenaphtenediimine

## Abstract

Complexes [(dpp-BIAN)^0^Co^II^I_2_]·MeCN (**I**) and [(Py)_2_CoI_2_] (**II**) were synthesized by the reaction between cobalt(II) iodide and 1,2-bis(2,6-diisopropylphenylimino)acenaphthene (dpp-BIAN) or pyridine (Py), respectively. The molecular structures of the complexes were determined by X-ray diffraction. The Co(II) ions in both compounds are in a distorted tetrahedral environment (CoN_2_I_2_). The electrochemical behavior of complex **I** was studied by cyclic voltammetry. Magnetochemical measurements revealed that when an external magnetic field is applied, both compounds exhibit the properties of field-induced single ion magnets.

## 1. Introduction

The activity in the research of mononuclear cobalt complexes with various types of donor ligands, which has been observed in the last decade, is explained by the orbital moment of the Co(II) ion as well as the spin-orbit coupling [1,2]. Both of these factors influence the possibility of splitting the term of the ground state of the complex in a zero field. Axial splitting in a zero field (D), which characterizes the magnetic anisotropy of the metal ion in the complex, is a necessary condition for the manifestation of the properties of a single molecule or single ion magnet (SIM). Creating conditions for a metal ion to manifest high magnetic anisotropy is a priority in the chemistry of complexes compared to additive schemes for increasing the total spin of the system. The anisotropy of the Co(II) ion can be controlled by rational selection of ligands with a set of finely tuned characteristics of the spatial and electronic structure. The use of spatially branched functional groups capable of defining unusual types of geometry of the Co(II) atom is effectively used in the design of SIMs. On the other hand, fine tuning of magnetic anisotropy can be carried out by varying the ligand field strength, which is usually achieved due to the optimal combination of the number and relative position of substituents of a different electronic nature. At the same time, the strategy of the controlled ligand field strength, based on the use of redox-active molecules capable of reversibly and controllably changing their electronic characteristics while maintaining a high degree of structural rigidity, has been studied scarcely for the molecular design of SIM [3,4,5].

Redox-active ligands can form metal-radical complexes, interest in which is caused when solving problems of creating materials with tuned properties [6,7]. The generation/degradation of the radical in the composition of the complex can significantly affect the luminescent and magnetic properties, conductivity, catalytic activity, etc. [8,9,10,11,12]. 

As the main object of our research, we selected a complex of cobalt(II) iodide with 1,2-bis(2,6-diisopropylphenylimino)acenaphthene (dpp-BIAN). The ligand dpp-BIAN is widely known for its unique ability to accept reversibly up to four electrons [13], which allows one to control the ligand field strength over a wide range. Moreover, the catalytic activity towards a number of industrially important reactions [14,15,16,17], visible light-harvesting behavior for potential photovoltaic applications [18,19], biological activity [20], as well as ability for small molecule activation [21] shown by transition metal complexes of neutral, mono-, and dianionic forms of dpp-BIAN could be finely tuned due to the unique redox features of this ligand [22,23,24,25,26,27,28]. On the other hand, the possibility of reversible generation of radical anions ([(dpp-BIAN)^0^]⇄[(dpp-BIAN)**^·^**^−^]) may allow the exchange interactions of the ferromagnetic nature in the complex molecule to be switched on, which can also have an effect on the SIM magnetization reversal barrier. Iodide anions were selected as additional ligands due to data indicating the heavy atoms effect of halogens on the magnetic anisotropy of Co(II)-based SIMs [29].

As an additional object of research, we studied the complex of cobalt(II) iodide with pyridine. Despite their chemical simplicity, it is surprising that the coordination compounds of CoI_2_ with substituted pyridine ligands have received scarce attention [30,31,32,33,34,35,36]. A comparison study of these two systems allowed us to draw preliminary conclusions about the potential use of redox-active ligands of the Ar-BIAN class in the Co(II)-SIM-directed design. 

## 2. Results and Discussion

### 2.1. Synthesis and Characterization of [(dpp-BIAN)^0^Co^II^I_2_]·MeCN (I) and [(Py)_2_CoI_2_] (II)

**Scheme 1:**
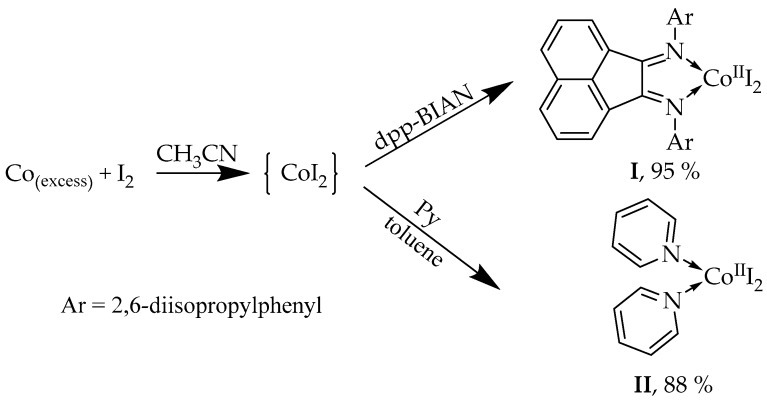
Synthesis of complexes **I** and **II**.

The reaction between 1,2-bis[(2,6-diisopropylphenyl)imino]acenaphthene and cobalt diiodide in a solution of acetonitrile (Scheme 1) resulted in complex [(dpp-BIAN)^0^Co^II^I_2_] (**I**). The reaction proceeded in an inert atmosphere on heating and was accompanied by a rapid change in the color of the solution from green to red-brown. The product crystallized at room temperature from the initial volume of solvent in high yield (95%). Due to the extreme hygroscopicity of cobalt iodide [37], it was synthesized in situ from crystalline iodine and an excess of metal cobalt. Preliminary data on the state of the redox-active ligand in **I** can be obtained from its IR spectrum by the presence of characteristic bands of stretching vibrations of the double bond C=N (1646–1600 cm^−1^) of neutral diimine dpp-BIAN [38,39] and the absence of absorption bands of the three-electron C‒N bonds (1550–1500 cm^−1^) of the dpp-BIAN anion-radical and the C‒N bonds (1310 cm^−1^) of the dianionic form of the ligand [40,41]. The synthesis of the cobalt(II) iodide complex with dpp-BIAN was first reported in an article on the polymerization of α-olefins [39], but its crystal structure was not described since single crystals were not isolated—the composition of the obtained compound was established by indirect methods. The synthesis and molecular structure of another congener [(mes-BIAN)^0^Co^II^I_2_] (mes-BIAN = di-(2,4,6-trimethylphenyl)-bis-acenaphthenequinonediimine) was reported; however, their magnetic and redox properties were not discussed [42].

When pyridine was allowed to react with cobalt(II) iodide in a 2:1 molar ratio in toluene (Scheme 1), complex [(Py)_2_CoI_2_] (**II**) was isolated. Dry CoI_2_ was used for the synthesis (see the experimental part), in which a toluene medium reacted heterogeneously with two equivalents of pyridine. Single crystals suitable for X-ray diffraction were grown by slowly (10 °C per hour) cooling the reaction mixture from 130 to 25 °C and then keeping it for 2 h. For the first time, the synthesis of the complex of cobalt diiodide with pyridine was described as early as 1927 [43]; the authors described the color of the compound (blue) and performed elemental analysis of the iodine content. In 1978, Bill Little et al. [44] described the synthesis of the compound of the composition [(Py)_2_CoI_2_] and characterized it by powder X-ray diffraction. Thus, single crystal X-ray diffraction studies for compound **II** were performed here for the first time. The powder X-ray diffraction pattern of the compound prepared here (Figures S1, S2, see Supplementary Material) differs from the powder X-ray diffraction pattern reported [44] (Table S1, see Supplementary Material). No similarity of interplanar distances was observed, which allows us to conclude that, apparently, in this work, we obtained another compound or another polymorphic modification of [(Py)_2_CoI_2_].

### 2.2. Molecular Structures

**Figure 1:**
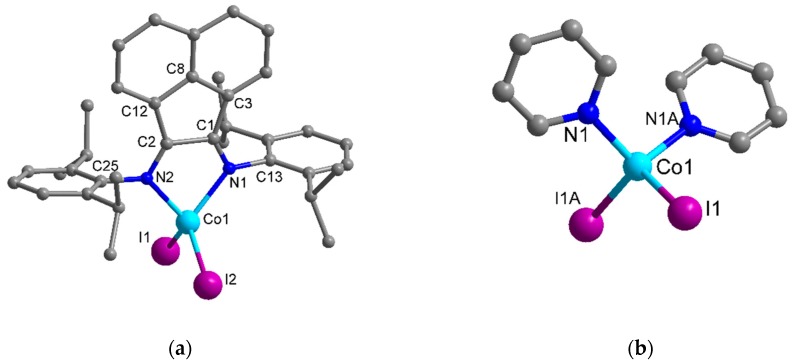
Molecular structure of complexes I (**a**) and II (**b**). Hydrogen atoms are omitted. Symmetry codes used to generate equivalent atoms for II: (A) −x + 1, y, −z + 1/2.

Complexes **I** and **II** crystallized in the orthorhombic space groups *Pbca* and *Pbcn*, respectively; the crystal and experiment data are listed in Table 1. The crystal of complex **I** is solvate with one molecule of MeCN. Both compounds are mononuclear, in which the cobalt(II) ion coordinates two I^−^ anions and two N atoms of chelate ligand dpp-BIAN (**I**, Figure 1a) or two monodentate ligands Py (**II**, Figure 1b). In crystal **II**, the second-order axis *C*_2_ passes through the metal atom between pairs of atoms I and N. For complex **I**, the N‒Co‒N angle (81.24(12)°) is much smaller than the others (N‒Co‒I 112.49(9)‒117.61(9)°, I‒Co‒I 109.33(2)°) due to the chelating nature of the ligand (Co‒N 2.073(3), 2.090(3) Å, Co‒I 2.5343(7), 2.5346(6) Å). In **II**, the I‒Co‒I angle (115.80(7)°) is larger, while the N‒Co‒I (108.0(2)°, 109.0(2)°) and N‒Co‒N (106.7(4)°) angles are close to the angle for a perfect tetrahedron. The Co‒N bond length (2.041(7) Å) in **II** decreased by ~0.04 Å as compared to **I**, while the Co‒I bond (2.5643(11) Å) increased by ~0.03 Å. The observed bond lengths are comparable with the known values for complexes with a similar coordination environment of CoN_2_I_2_ [29,32,35,36,39,45,46]_._ The deviation of the angular geometry of the polyhedra from the ideal tetrahedron was estimated by calculating the parameter δ = 2*T*_d_ − (α + β), where angles α = N‒Co‒N, β = I‒Co‒I, and *T*_d_ = 109.5°. Based on the calculations, the coordination environment of CoN_2_I_2_ in **I** corresponds to a compressed pseudotetrahedron (δ = 28.43), while that in **II** corresponds to a tetrahedron (δ = −3.5).

The C−C and C−N bond lengths in the α-diimine fragment of complex **I** (C(1)−C(2) 1.515(5) Å, C(1)−N(1) 1.276(5) Å, C(2)−N(2) 1.280(5) Å)) as compared to dpp-BIAN (C−C 1.527 Å, C−N 1.272, 1.272 Å) [47] as well as cobalt(II) [(dpp-BIAN)^0^Co^II^Cl_2_] (C−C 1.564 Å, C−N 1.231, 1.286 Å) [39,48], [(dpp-BIAN)^0^Co^II^Br_2_] (C−C 1.531 Å, C−N 1.268, 1.286 Å) [49], and iron(II) [(dpp-BIAN)^0^Fe^II^Br_2_] complexes (C−C 1.508 Å, C−N 1.277, 1.287 Å) [50], [(dpp-BIAN)^0^Fe^II^Cl_2_] (C−C 1.506 Å, C−N 1.288, 1.271 Å) [51] indicate the neutral form of the ligand. 

In **I**, π–π interactions were observed between the benzene rings of the acenaphthene fragment (the shortest C...C distance is 3.308(6) Å), the interatomic distance between nearby Co atoms is 9.7904(2) Å. In the crystal of molecule **II**, π–π interaction was observed between parallel pyridine ligands of neighboring complex molecules (the shortest C...C distance is 3.522(14) Å); the minimum interatomic Co...Co distance is 7.6327(9) Å. An increase in the organic component decreases the calculated crystal density from 2.224 (**II**) to 1.515 g/cm^3^ (**I**).

### 2.3. Solid State Magnetic Susceptibilities

**Figure 2:**
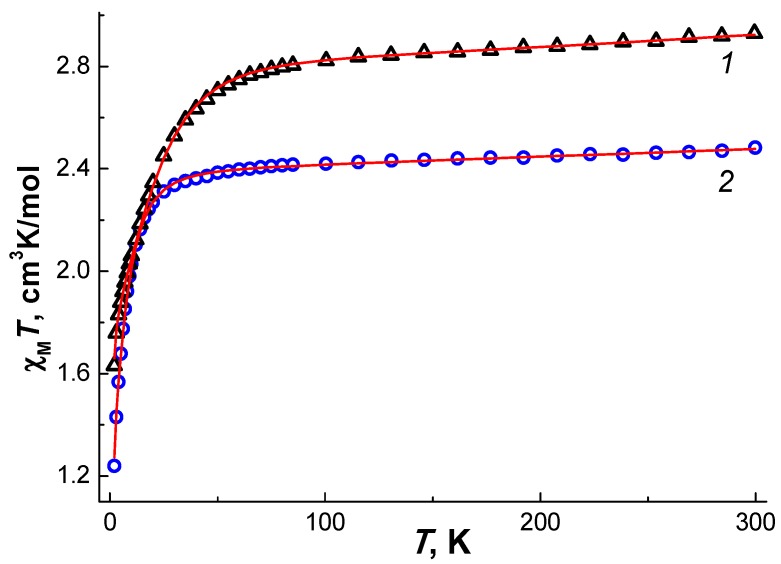
Temperature dependences χ_m_*T* of complexes **I** (*1*) and **II** (*2*) (*H* = 5 kOe). Lines are the result of approximation of experimental data with Equation (1) (see text).

The temperature dependences of the magnetic susceptibility of complexes **I** and **II** were studied in the range of 2–300 K in a magnetic field of 5 kOe (Figure 2). The χ_m_*T* values of both complexes at 300 K (2.93 and 2.48 cm^3^ mol^−1^ K for **I** and **II**, respectively) are significantly higher than the pure spin value (1.88 cm^3^ mol^−1^ K) [52], which may indicate a significant orbital contribution to the total magnetic moment. The χ_m_*T* dependence remains almost linear to 100 K; with a further decrease in temperature, the χ_m_*T* values gradually decrease and reach a minimum at *T* = 2 K (1.63 and 1.24 cm^3^ mol^−1^ K for **I** and **II**, respectively). Such behavior may be due to significant magnetic anisotropy, and/or the Zeeman effect (saturation effect) when an external magnetic field is applied.

The χ*T* curves were approximated using spin Hamiltonian (Equation (1)):

(1) $$\hat{H}=D\left[{\hat{S}}_{Z}^{2}-\frac{1}{3}S\left(S+1\right)\right]+\mu_{B}\left(B_{X}g_{X}{\hat{S}}_{X}+B_{Y}g_{Y}{\hat{S}}_{Y}+B_{Z}g_{Z}{\hat{S}}_{Z}\right)$$

where S = 3/2 is the spin of the high-spin Co(II) ion, D is the axial ZFS parameter, and g_α_ (α = X, Y, Z) are the principle values of the g-tensor; $\mu_{B}$ is the Bohr magneton; and *g*_x_, *g*_y_, and *g*_z_ are the components of the *g*-tensor using the PHI program [53]. An analysis of the approximation results revealed the presence of an excessive number of parameters in the case of an anisotropic *g*-tensor; therefore, the case of an isotropic *g*-tensor (*g*_x_ = *g*_y_ = *g*_z_ = *g*_iso_) was used. Our calculations (see Table 2) showed that the curves can be described by both negative and positive parameters *D*. Nonzero absolute values of *D* indicate the presence of magnetic anisotropy of the cobalt(II) ion. A nonzero negative value of the parameter *zJ*’ for **II** indicates the presence of weak intermolecular interactions between metal ions, which correlates with the X-ray diffraction data.

*CASSCF/NEVPT2* calculations allowed this ambiguity to be passed by determination of the zero-filed splitting parameters for cobalt(II) ions in **I** and **II**. The calculated principle values of the *D*- and *g*-tensors for **I** and **II** are listed in Table 2, where all the calculated *D* values are negative, and the *D* and *g*_iso_ values are all close to the values calculated for the experimental magnetic data. The negative values of the ZFS parameters suppose easy-axis magnetic anisotropy and the probability of realizing SIM properties (out-of-phase AC susceptibility signals and slow magnetic relaxation) [29,54].

Table 3 displays the energy levels (cm^−1^) for **I** and **II** calculated from spin Hamiltonian (1) and *CASSCF/NEVPT2* (in zero-filed and applied field). The energy values fitted and calculated form *CASSCF/NEVPT2* are in good agreement.

In order to identify SIM behavior, measurements of the AC magnetic susceptibility were performed. The out-of-phase signal χ’’ close to zero was observed in the absence of the DC-magnetic field for both complexes. An external DC-magnetic field *H*_dc_ application made it possible to detect distinct maxima on the χ’’ values at 2 K in both cases. The optimal values of *H*_dc_ (1.5 and 2.5 kOe for **I**·MeCN and **II**, respectively) were obtained by the AC susceptibility measurements at various DC field strengths (Figures S3 and S4, see Supplementary Material).

**Figure 3:**
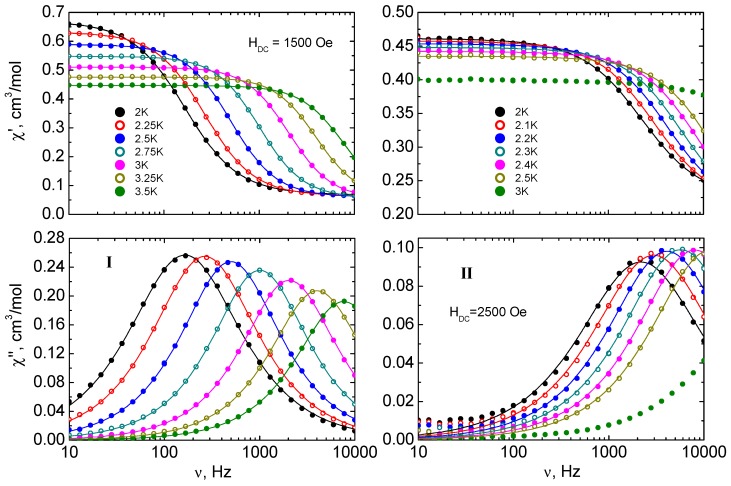
Frequency dependences of the real (χ’, top) and imaginary (χ", bottom) parts of the AC magnetic susceptibility at different temperatures for complexes **I** (left, *H* = 1.5 kOe, *T* = 2‒3.5 K) and **II** (right, *H* = 2.5 kOe, *T* = 2‒3 K). Lines show approximations of the data by the generalized Debye model.

**Figure 4:**
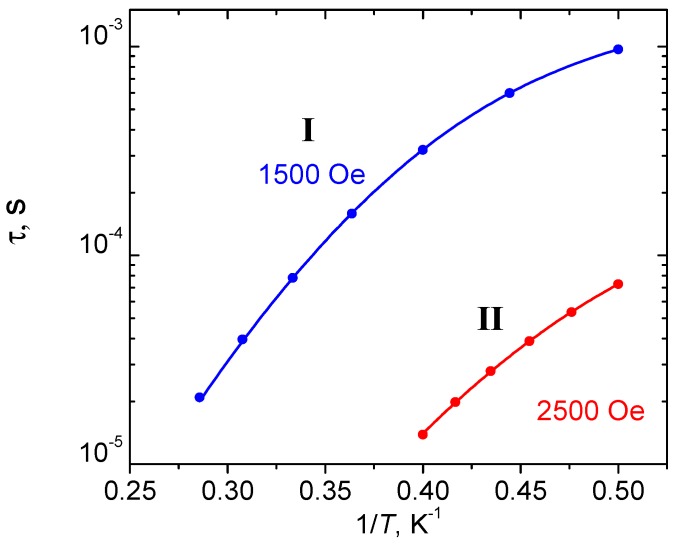
Dependence of the relaxation time τ on the reciprocal temperature for **II** (*H* = 2.5 kOe, *T* = 2‒2.5 K). Lines show approximations of the curves by the sum of the Raman and direct relaxation mechanisms (blue solid line).

The AC susceptibility data in the optimal DC field were fitted by the generalized Debye model (Figure 3), which allows determination of the relaxation time temperature dependencies of the magnetization (Figure 4). We tried to fit the τ(1/*T*) by the different relaxation mechanisms in order to better understand the relaxation pathways’ nature [55,56]. As we can conclude from this experiment, the best agreement between the experimental data and theoretical dependences was achieved using the sum of Raman (τ_Raman_^−1^ = *C*_Raman_*T*^n_Raman^) and direct (τ_direct_^−1^ = *A*_direct_*H*^4^*T*) relaxation mechanisms for both complexes (Figure 4, and Supplementary Material) with the best-fit parameters: *A*_direct_ = 7.4 and 7.5 ∙ 10^−11^ K^−1^Oe^−4^s^−1^, *C*_Raman_ = 0.46, and 12 s^−1^ K^-n_Raman^, *n*_Raman_ = 9.2 and 9.4 for **I** and **II**, respectively.

The fitting of the field dependences of the relaxation time as well as the negligible out-of-phase susceptibility in a zero field gives clear evidence of some additional temperature-independent relaxation pathways, presumably, the quantum tunneling, which suppress slow magnetic relaxation (see Supplementary Material). The data fit with use of the quantum tunneling (τ_QTM_^−1^ = *B*_1_/(1+*B*_2_*H*^2^)) as an additional relaxation mechanism leads to overparameterization and mutual dependencies of the fit parameters.

It should be noted that the amplitude of the signal χ’’(ν) for complex **II** in the optimal field (Figure 3) increases with the temperature, which may be due to intermolecular or dipole–dipole interactions present in this compound. A similar behavior was previously observed for cobalt complexes [57], as well as for other anisotropic ions, in particular, lanthanide ions [34,58,59,60]. For complex **I**, this behavior was not observed, which may be due to the large distance between metal ions in the crystal lattice.

A comparative analysis of the properties of **I** and similar complexes with chelate ligands (2,2’-biquinoline (biq) [34], 4,7-diphenyl-2,9-dimethyl-1,10-phenanthroline (bcp) [35], and 2,9-dimethyl-1,10-phenanthroline (dmphen) [36] showed that the value of the parameter *D* can be both positive (10.3 cm^−1^ for [CoI_2_(biq)] and 16.6 cm^−1^ for [CoI_2_(dmphen)]) and negative (−7.03 cm^−1^ for [CoI_2_(bcp)]).

Complex **II** can be compared with analog [CoI_2_(quinoline)_2_] [29], for which *D* = 9.2 cm^−1^ and there is no slow magnetic relaxation. So, the nature of pyridine ligand changes the electronic structure of cobalt(II) ions, and this fine chemical tuning results in a switch of the magnetic behavior at low temperatures.

### 2.4. Cyclic voltammetry of [(dpp-BIAN)^0^Co^II^I_2_]·MeCN (I)

**Figure 5:**
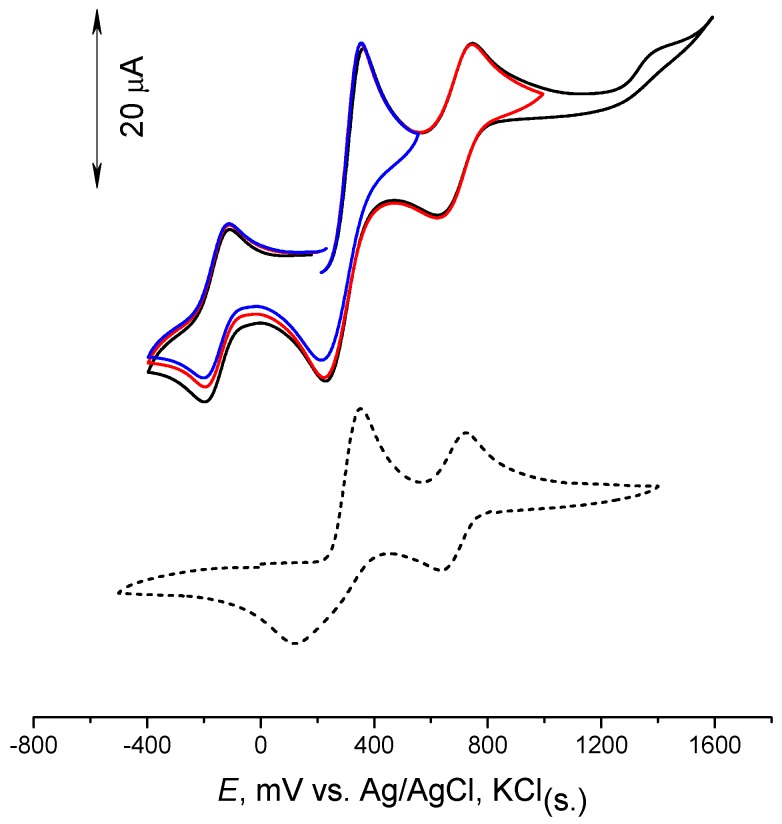
Voltammogram of **I** (solid lines, 2.1 mM at 0.05 M *n*-Bu_4_NBF_4_ in MeCN, 50 mV/s, Pt) and Bu_4_NI (dashed line).

The electrochemical behavior of complex **I** was studied in solution; voltammograms were recorded for 2.1 M solutions of **I** in a 0.05 M solution of *n*-Bu_4_NBF_4_ in acetonitrile on a platinum disk working electrode (Figure 5).

When dissolved in acetonitrile, both iodine atoms at the cobalt atom are replaced by solvate acetonitrile molecules. This follows from the analysis of the voltammograms with a potential sweep into the anode region (Figure 5): The first two anode processes correspond to the oxidation of free iodide ions (I^−^/I_3_^−^ and I_3_^−^/I_2_ at potentials *E*_pa_ = 0.36 V and 0.75 V, respectively). This is followed by an irreversible redox transition (*E*_pa_ = 1.39 V at 50 mV/s), which corresponds to the oxidation of the ligand in the complex. This was concluded based on a comparison with the literature data for the imine oxidation potentials. The imine oxidation is irreversible and is observed in the range 1.5–1.7 V: For example, for PhCH=NPh, the oxidation potential is 1.59 V (vs SCE, Pt, MeCN [61]); and for free dpp-BIAN, the oxidation potential is 1.02 V (Pt, THF, vs. Fc^+/0^ when converted into Ag/AgCl, KCl_sat_, this is 1.47 V [62]).

**Scheme 2:**
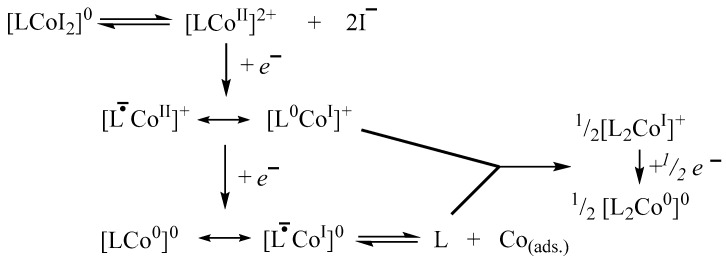
Cathodic transformations of complex **I**.

In the cathode potential region, a quasi-reversible redox transition **I** was observed with the structure of **[L^0^Co^I^]^+^** to the electronic structure of the reduced complex (Scheme 2).

**Figure 6:**
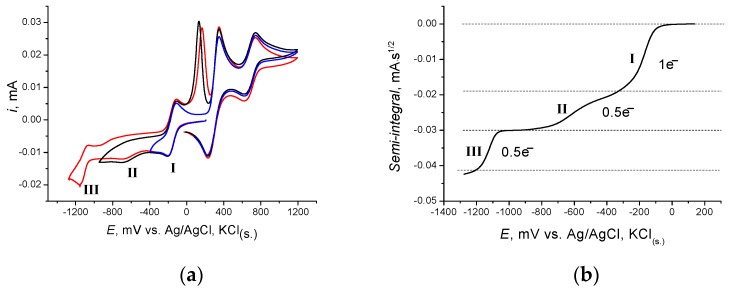
(**a**) Voltammogram of **I** (2.1 mM in 0.05 M *n*-Bu_4_NBF_4_ in MeCN, 50 mV/s, Pt); (**b**) semi-integral transformation of the cathode part of the voltammogram shown in Figure 6a.

At a potential of −0.68 V, a cathodic peak **II** was observed accompanied by the destruction of the complex with the deposition of cobalt on the electrode surface, which can be seen from the appearance of the oxidation peak of cobalt deposited on the backward curve (*E*_pa_ = +0.13 V) [63]. This cathodic process proceeds electrochemically slowly, which manifests itself in a very flat peak shape (*E_p_ − E_p/2_* = 93 mV). In addition, analysis of the semi-integrated form of the voltammogram indicates that approximately half of the total amount of the complex is reduced (Figure 6b). It seems that free dpp-BIAN released upon cathodic destruction binds **[L^0^Co^I^]^+^** to form a new complex [**L_2_^0^Co^I^]^+^**. It contains a greater number of donor ligands than the original, which complicates its reduction. It is observed only when potential *E*_1/2_ = −1.11 V (peak **III**), which is substantially more anodic than the reduction potential of free ligand dpp-BIAN (−1.56 V [64]), which confirms the assignment of this redox transition. The limiting value of the half-integral function for the first cathode transition is equal to the sum for the second and third ones, which corresponds to the stoichiometry proposed in Scheme 2.

## 3. Materials and Methods 

### 3.1. General Remarks

Initial cobalt(II) iodide is sensitive to air moisture (it forms crystalline hydrates); therefore, all synthetic procedures and the isolation of reaction products were performed in an inert atmosphere using the Schlenk technique. Acetonitrile was dried over phosphorus(V) oxide and stored over molecular sieves (3 Å), toluene was dried and stored over the sodium complex with benzophenone, and the solvents were taken using condensation in vacuum immediately before use. Cobalt(II) iodide was synthesized *in situ* from metallic cobalt and crystalline iodine (Merck KGaA, Darmstadt, Germany) in acetonitrile in a glass ampoule with a Teflon valve under heating; dpp-BIAN was synthesized according to the known procedure [38]; and pyridine (Merck KGaA, Darmstadt, Germany) was dried over potassium hydroxide, stored over activated molecular sieves (4 Å), and condensed in vacuum immediately before the reaction. Product yields were calculated relative to the initial amount of ligands: dpp-BIAN (0.50 g, 1 mmol) and pyridine (0.158 g, 2 mmol).

IR spectra of the compound were recorded in the range 400–4000 cm^−1^ on a Perkin Elmer (Waltham, Massachusetts, USA) Spectrum 65 spectrophotometer equipped with Quest ATR Accessory (Specac (Orpington, Kent, UK)) using the method of attenuated total reflection (ATR).

Voltammograms of the complexes were recorded on an AutoLab PGSTAT100 N (Metrohm, Zurich, Switzerland) potentiostat in a 10-mL three-electrode cell with a platinum wire counter electrode and a silver reference electrode (Ag/AgCl, KCl_sat_). A reference electrode was attached to the electrolyte solution through a salt bridge containing a 0.05 M solution of *n*-Bu_4_NBF_4_ in acetonitrile. A platinum disk with an active surface area of 0.045 cm^2^ was used as a working electrode. The platinum electrode was polished with a suspension of Al_2_O_3_ (SPA 0.3) at a polishing set (Metrohm, Zurich, Switzerland) and washed with sulfuric acid and water with acetone. All solutions were thoroughly deaerated with argon. A solution of complex **I** was prepared in acetonitrile in a Schlenk vessel in an argon atmosphere.

The magnetic behavior of the complexes was studied using the automated Quantum Design PPMS-9 physical property measuring system with the option of measuring the dynamic (AC) and static (DC) magnetic susceptibility. This equipment allows research to be carried out in the temperature range of 2–300 K with magnetic fields from −9 to 9 T. When measuring the AC susceptibility, an alternating magnetic field was applied with intensity *H*_AC_ = 1–5 Oe in the frequency range 10–10000 Hz. The measurements were carried out on polycrystalline samples moistened with mineral oil to prevent the orientation of the crystals in an external magnetic field. The prepared samples were sealed in plastic bags. The magnetic susceptibility χ was determined taking into account the diamagnetic contribution of the substance, using the additive Pascal scheme, the contribution of the bag, and that of mineral oil. The magnetization relaxation times τ = 1/2πν_max_ and the α factor that determines the distribution of relaxation processes were calculated by approximating the dependences χ′(ν) and χ″(ν) by the generalized Debye model.

Elemental analysis was performed on an automatic EuroEA-3000 C, H, N, S analyzer (EuroVektor, Pavia, Italy).

X-ray powder diffraction studies were carried out on a Bruker D8 Advance powder X-ray diffractometer (Bruker AXS, Madison, Wisconsin) equipped with variable slots, an Ni filter, and a LynxEye position-sensitive detector. The survey was carried out in the X-ray reflection mode (Bragg-Brentano geometry) with rotation of the sample. The diffraction pattern was described by the Rietveld method in the TOPAS software package.

X-ray diffraction studies of single crystals of complexes **I** and **II** were performed on a Bruker Apex II diffractometer (Bruker AXS, Madison, Wisconsin) (CCD detector, Mo*K*_α_, λ = 0.71073 Å, graphite monochromator) [65]. The structures were solved by direct methods and refined in the full-matrix anisotropic approximation for all non-hydrogen atoms. Hydrogen atoms at carbon atoms of organic ligands were generated geometrically and refined in the "riding" model. The calculations were performed using the SHELX-2014 software package [66]. The main crystallographic parameters and refinement details of compounds **I** and **II** are listed in Table 1. The structure parameters were deposited with the Cambridge Structural Database (CCDC Nos. 1961151 (**I**) and 1961152 (**II**); deposit@ccdc.cam.ac.uk or http://www.ccdc.cam.ac.uk/data_request/cif).

Quantum chemical calculations of the zero-field splitting (ZFS) parameters for complexes **I** and **II** were performed based on the state-averaged complete active space self-consistent field calculations (SA-CASSCF) [67,68,69], followed by the N-electron valence second-order perturbation theory (NEVPT2) [70,71]. Scalar relativistic effects were taken into account by a standard second-order Douglas–Kroll–Hess (DKH) procedure [72]. For the calculations, a segmented all-electron relativistically contracted version [73] of Ahlrichs polarized triple-ζ basis set def2-TZVP [74,75,76] was used for all atoms. In order to decrease the calculation time, the resolution of identity (RI) approximation with corresponding correlation fitting of the basis set [77] was employed. The spin–orbit effects were included using the quasi-degenerate perturbation theory (QDPT) [78] in which approximations to the Breit–Pauli form of the spin–orbit coupling operator (SOMF approximation) [79] and the effective Hamiltonian theory [80] were utilized. The CASSCF active space is defined by considering the seven d-electrons (for Co^II^) distributed in the five 3d orbitals. All possible multiplet (10 quartet and 40 doublet) states arising from the d^7^ configuration were included into the wave function expansion. All quantum chemical calculations were performed by the *ORCA* program (ver.4.2.0) [81].

### 3.2. Synthesis of [(dpp-BIAN)^0^Co^II^I_2_]·MeCN (**I**)

Ligand dpp-BIAN (0.5 g, 1 mmol) was added to cobalt diiodide (1 mmol) prepared *in situ* in acetonitrile from iodine (0.254 g, 1 mmol) and an excess of powder metal cobalt. The color of the reaction mixture instantly changed from green to red-brown. Crystallization from acetonitrile gave red crystals in the form of parallelepipeds. Yield, 0.813 g (95%). Anal. calcd. for C_38_H_43_CoI_2_N_3_ (%): C, 53.41; H, 5.07; N, 4.92. Found (%): C, 53.35; H, 4.93; N, 4.87. IR (ATR, ν, cm^−1^): 3063 w, 2961 s, 2925m, 2865m, 2249w, 1646m, 1620s, 1600m, 1583s, 1489w, 1462s, 1436s, 1420m, 1383m, 1364m, 1323m, 1291s, 1253m, 1222m, 1203w, 1188m, 1128m, 1112w, 1089w, 1052m, 1042m, 953m, 936m, 852w, 833s, 800vs, 779vs, 757vs, 738m, 611w, 587w, 541s, 515w, 468m, 436w.

### 3.3. Synthesis of [(Py)_2_CoI_2_] (II)

Cobalt diiodide (1 mmol) was synthesized *in situ* from crystalline iodine (0.254 g, 1 mmol) and an excess of powder metal cobalt in acetonitrile. Acetonitrile was removed from the {CoI_2_ **·** *x*MeCN} adduct by drying in dynamic vacuum at 120 °C for 3 h. Toluene (25 mL) was condensed into a vial with CoI_2_; then, pyridine (0.158 g, 2 mmol) was added, and the vial was sealed and heated in an oil bath at 130 °C until CoI_2_ was completely dissolved. The color of the reaction mixture was blue. Slow cooling (10 °C per h) led to the formation of blue needle-shaped single crystals suitable for X-ray diffraction. Yield, 0.415 g (88%). Anal. calcd. for C_10_H_10_CoI_2_N_2_ (%): C, 25.50; H, 2.14; N, 5.95. Found (%): C, 25.28; H, 2.08; N, 5.74. IR (ATR, ν, cm^−1^): 3058w, 1859w, 1660w, 1604vs, 1482m, 1442vs, 1352w, 1241w, 1208s, 1154w, 1061vs, 1044m, 1014m, 945w, 756vs, 691vs, 642vs, 446w, 426s, 416w.

## 4. Conclusions

Thus, here we presented the synthesis, characterization, and structures of two molecular complexes [(dpp-BIAN)^0^Co^II^I_2_]·MeCN (**I**) and [(Py)_2_CoI_2_] (**II**). The electrochemical studies of **I** suggest one-electron reduction of the ligand in the composition of the complex to a radical, which is useful for further study of its magnetic characteristics. The results of the *DC* magnetic measurements as well as *CASSCF/NEVPT2* calculations showed that the distortion of the coordination CoN_2_I_2_ environment results in a negative axial magnetic anisotropy decrease from **II** (*D* = −5.9 cm^−1^) to **I** (*D* = −22.6 cm^−1^). According to the AC magnetometry, the complexes studied exhibit superparamagnetism and slow magnetic relaxation in the applied field *H_DC_*, which classifies these compounds as field-induced SIMs. By the precise fit of the relaxation time dependencies it was shown that the main pathways of relaxation are Raman and direct, which is in common with other reported tetrahedral Co(II) SIMs.

## Figures and Tables

**Table 1:** Crystallographic data, details of the data collection, and characteristics of the data refinement for **I** and **II.**

Parameter	Value	
	I	II
Molecular formula	C_38_H_43_CoI_2_N_3_	C_10_H_10_CoI_2_N_2_
*Mw*	854.48	470.93
*T,* K	296(2)	
Crystal system	Orthorhombic	
Space group	*Pbca*	*Pbcn*
*a*, Å	19.5759(4)	13.488(2)
*b*, Å	19.3545(4)	7.1497(10)
*c*, Å	19.7703(5)	14.588(2)
*V*, Å^3^	7490.6(3)	1406.7(3)
*Z*	8	4
ρ_calcd_, g cm^−3^	1.515	2.224
μ, mm^−1^	2.135	5.582
θ range, deg	2.06–28.28	2.79–30.59
*F*(000)	3400	868
Index range	−26 ≤ *h* ≤ 25; −25 ≤ *k* ≤ 25; −26 ≤ *l* ≤ 25	−19 ≤ *h* ≤ 19; −10 ≤ *k* ≤ 10; −20 ≤ *l* ≤ 20
Number of reflections collected	75256	15566
Number of unique reflections	9291	2160
*R*_int_	0.099	0.162
Number of reflections with *I* > 2σ(*I*)	6692	1030
*GooF*	1.051	1.000
*R* factor on *F*^2^ > 2σ(*F*^2^)	*R*_1_ = 0.062, *wR*_2_ = 0.140	*R*_1_ = 0.074, *wR*_2_ = 0.148
*R* factor (all data)	*R*_1_ = 0.091, *wR*_2_ = 0.155	*R*_1_ = 0.157, *wR*_2_ = 0.182
Δρ_max_/Δρ_min_, *e*/Å^3^	−1.113/0.837	−1.135/0.966

**Table 2:** Scalar *D*- and *g*-tensor parameters fitted with Equation (1) and *CASSCF/NEVPT2* calculation for **I** and **II.**

Complex/ Parameter	Best-Fit Parameters with Equation (1)			CASSCF/NEVPT2 Calculation		
	*D*, cm^−1^	*g*_iso_	*zJ*, cm^−1^	*D*, cm^−1^	*E/D*	*g*
**I**	−25.7(3)	2.481(1)	0	−22.6	0.294	*g*_x_ = 2.251 *g*_y_ = 2.331 *g*_z_ = 2.515 (*g*_iso_ = 2.366)
	24.6(3)	2.481(1)	0			
**II**	−8.2(2)	2.285(1)	−0.07(1)	−5.9	0.094	*g*_x_ = 2.248 *g*_y_ = 2.272 *g*_z_ = 2.333 (*g*_iso_ = 2.284)
	7.6(2)	2.287(1)	−0.09(1)			

**Table 3:** Energy levels (cm^−1^) for **I** and **II** from Hamiltonian (1) approximation of the temperature dependences and *CASSCF*/*NEVPT2* calculations.

State	*M*_s_	I (calc)	I (*H*_DC_ = 0 Oe)	I (*H*_DC_ = 1.5 kOe)	II (calc)	II (*H*_DC_ = 0 Oe)	II (*H*_DC_ = 2.5 kOe)
1	−3/2	0	0	0	0	0	0
2	+3/2	0	0	0.53	0	0	0.92
3	−1/2	50.58	51.4	51.58	11.98	16.40	16.67
4	+1/2	50.58	51.4	51.75	11.98	16.40	16.94

## Supplementary Materials

The following are available online at https://www.mdpi.com/1420-3049/25/9/2054/s1, Figures S1 and S2: Theoretical and experimental X-ray powder diffraction patterns of complexes **I** and **II**; Figures S3 and S4: Frequency dependences of the real (χ’) and imaginary (χ") parts of the dynamic magnetic susceptibility at different applied magnetic fields for complexes **I** and **II**; Table S1: Comparison of the position of the peaks in X-ray powder diffraction patterns with literature data for complex **II**. The relaxation time temperature dependencies fitted by using different mechanisms.
