# Supplementary material for: Complexes of Cobalt(II) Iodide with Pyridine and Redox Active 1,2-Bis(arylimino)acenaphthene: Synthesis, Structure, Electrochemical, and Single Ion Magnet Properties
Source: Molecules. 2020 Apr 28;25(9):2054. doi: 10.3390/molecules25092054 (PMC7249109; doi:10.3390/molecules25092054)
Supplement: Supplementary file 1 [file molecules-25-02054-s001.pdf]

Supplementary Materials

Complexes of Cobalt(II) Iodide with Pyridine and Redox Active 1,2-Bis(arylimino)acenaphthene: Synthesis, Structure, Electrochemical, and Single Ion Magnet Properties

Dmitriy S. Yambulatov [1,*], Stanislav A. Nikolaevskii [1,*], Mikhail A. Kiskin [1], Tatiana V. Magdesieva [2], Oleg A. Levitskiy [2], Denis V. Korchagin [3], Nikolay N. Efimov [1], Pavel. N. Vasil'ev [1], Alexander S. Goloveshkin [4], Alexey A. Sidorov [1] and Igor L. Eremenko [1,4]

1 N. S. Kurnakov Institute of General and Inorganic Chemistry, Russian Academy of Sciences, 31 Leninsky prosp., 119991 Moscow, Russian Federation

2 Lomonosov Moscow State University, Dept. of Chemistry, Leninskie Gory 1/3, Moscow, 119991, Russia

3 Institute of Problems of Chemical Physics, Russian Academy of Sciences, Chernogolovka, Moscow Region, 142432, Russia

4 Nesmeyanov Institute of Organoelement Compounds, Moscow, 119991, Russia

* Correspondence: yambulatov@yandex.ru (D.S.Y.); sanikol@igic.ras.ru (S.A.N.); Tel.: +7-495-955-4817 (S.A.N.)

The obtained compounds were studied by X-ray powder diffraction, and the data obtained allowed us to confirm their correspondence to the structures of the corresponding single crystals (Figures S1, S2).

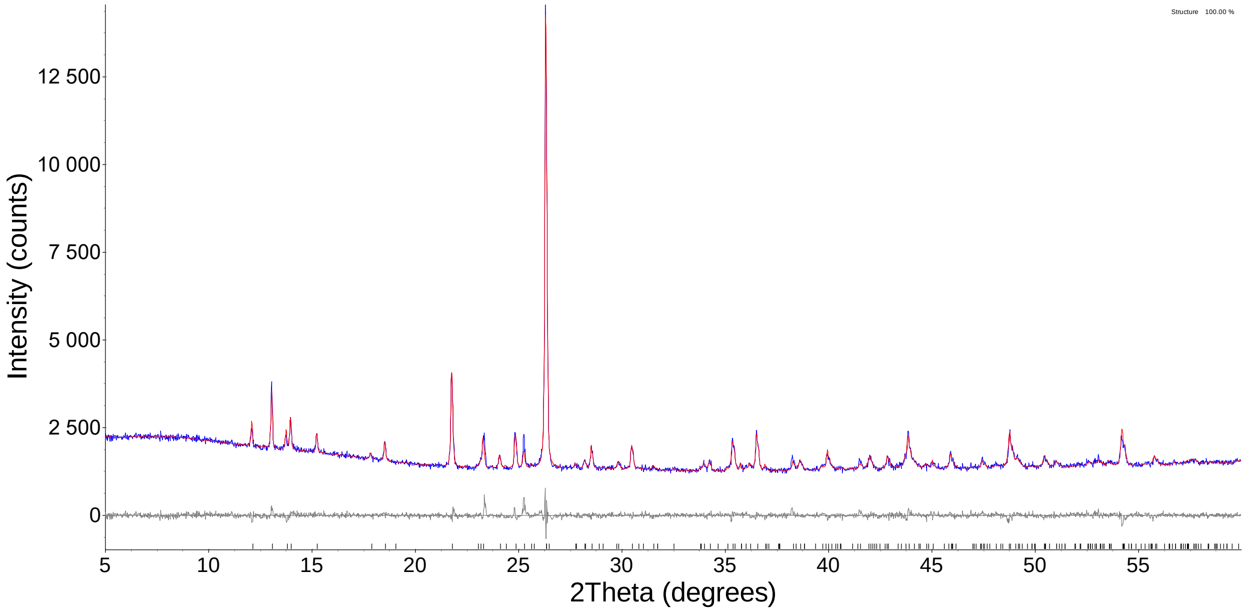

Figure S1. Theoretical (red) and experimental (blue) X-ray powder diffraction patterns of sample $[(dpp\text{-}BIAN)^0Co^{II}I_2]$ (**I**) and their difference (gray curve). Blue strokes show the estimated position of the lines.

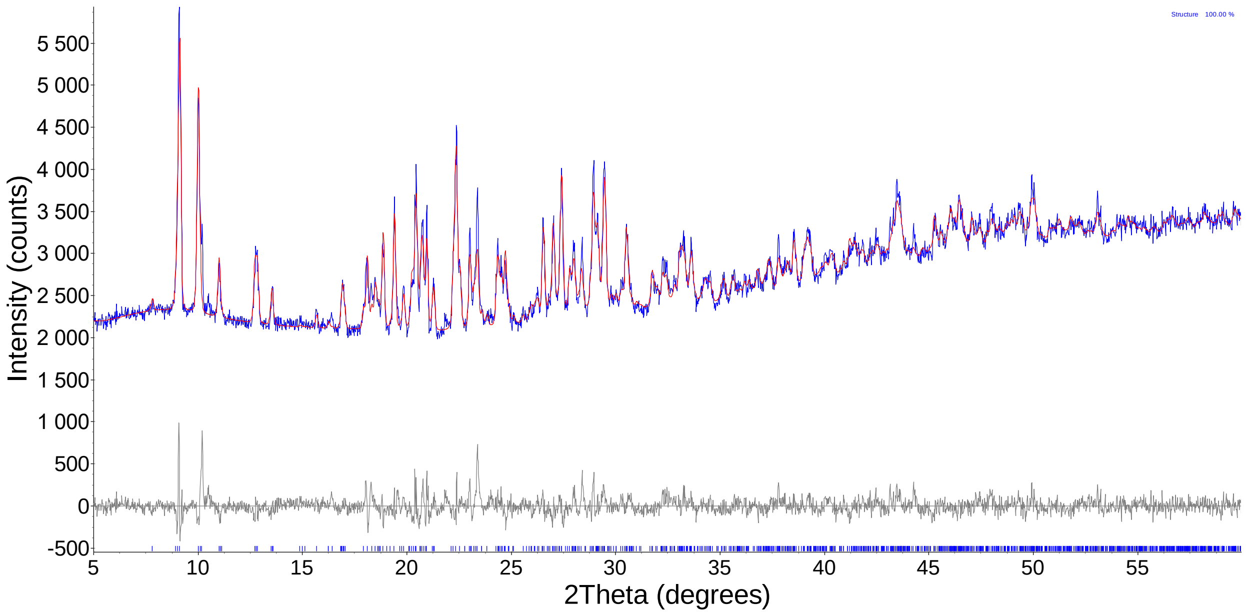

Figure S2. Theoretical (red) and experimental (blue) X-ray powder diffraction patterns of sample $[(Py)_2CoI_2]$ (**II**) and their difference (gray curve). Blue strokes show the estimated position of the lines.

Table S1 presents the characteristic intense peaks observed in the diffraction pattern of compound **II** at small diffraction angles ($2\theta < 20°$); their interplanar distances, and Miller indices are given. The interplanar distances reported in [1] are shown in the right column. Since the relative intensities are not given in [1], only the set of first peaks can be compared. As can be seen from Table S1, interplanar distances differs significantly.

1. *Little B.F., Long G.J.* // Inorg. Chem. V.17. 1978. P.3401–3413. doi: 10.1021/ic50190a021.

Table S1. Comparison of the position of the peaks in X-ray powder diffraction patterns with literature data.

2θ, deg	*d*, Å	*h*	*k*	*l*	*d*, Å [1]
9.02	9.80	2	0	0	9.17
9.13	9.68	0	2	0	8.21
10.01	8.83	1	0	2	6.43
10.16	8.70	0	2	1	5.50
11.00	8.03	1	1	2	4.54
12.71	6.96	2	0	2	4.28
12.78	6.92	0	2	2	
12.84	6.89	2	2	0	
13.56	6.52	1	2	2	
16.88	5.25	3	1	2	
16.96	5.22	3	2	1	
18.09	4.90	4	0	0	
18.31	4.84	0	4	0	
18.49	4.80	1	0	4	
18.61	4.76	2	2	3	
18.86	4.70	0	4	1	
19.40	4.57	1	4	1	
19.84	4.47	3	3	1	

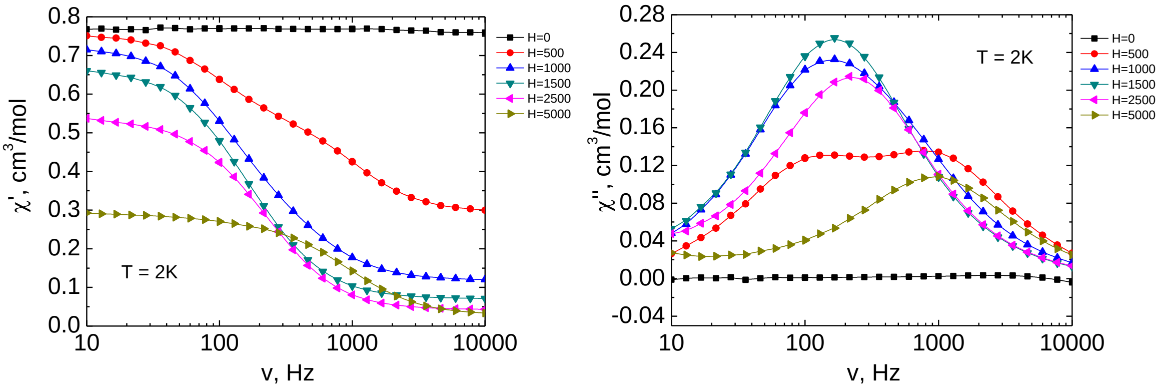

Figure S3. Frequency dependences of the real (χ', *left*) and imaginary (χ'', *right*) parts of the dynamic magnetic susceptibility at different applied magnetic fields for sample [(dpp-BIAN)^{0}Co^{II}I$_2$] (**I**). The lines are visual clues.

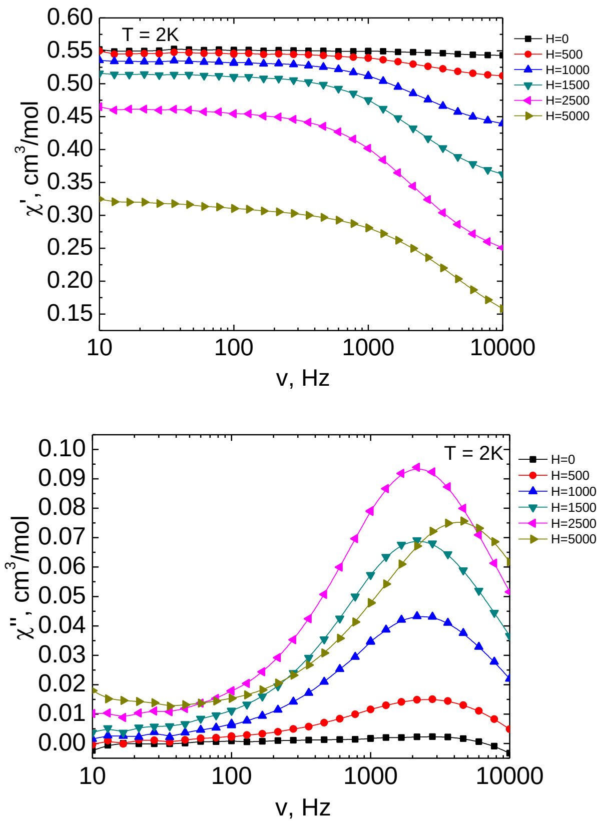

Figure S4. Frequency dependences of the real (χ',*top*) and imaginary (χ'',*bottom*) parts of the dynamic magnetic susceptibility at different applied magnetic fields for sample $[(Py)_2CoI_2]$ (**II**). The lines are visual clues.

The relaxation time temperature dependencies' fitting by the use of different mechanisms.

In order to better understand the possible relaxation pathways in complex under consideration, we tried to fit the temperature using the different fit function.

<table>
<tr><td>Dependence of the relaxation time τ on the reciprocal temperature for I (H = 1.5 kOe, T = 2–3.5 K).</td><td>Fit function, temperature range, and the best-fit parameters with uncertainties.</td></tr>
<tr><td>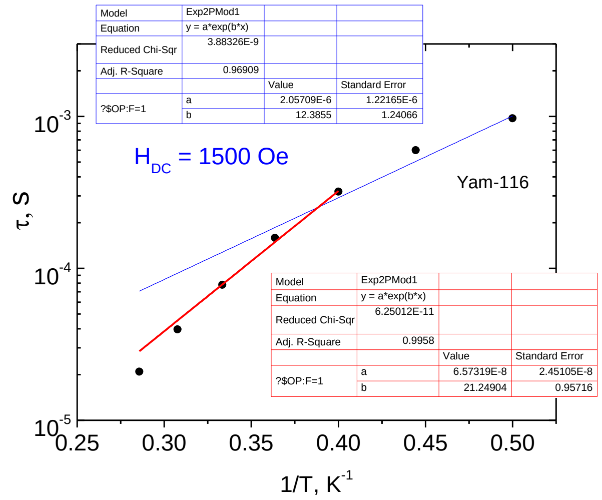
</td><td>

Orbach

$\tau = \tau_0 \cdot \exp\{\Delta E/kT\}$

T = 2–3.5 K
$\Delta E/k$ = 12.4±1.2 K
$\tau_0 = 2.1\cdot10^{-6} \pm 1.2\cdot10^{-6}$ s
R^2 = 0.9691 (blue line)

T = 2.5-3.5 K
$\Delta E/k$ = 21.2±1.0 K
$\tau_0 = 6.6\cdot10^{-8} \pm 2.5\cdot10^{-8}$ s
R^2 = 0.9958 (red line)

</td></tr>
<tr><td>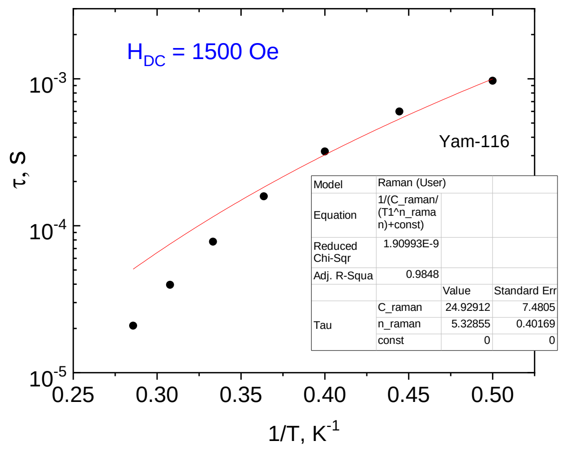
</td><td>

Raman

$\tau^{-1} = C_{Raman} T^{n\_Raman}$

T = 2–3.5 K
C_{Raman}= 24.9±7.5 $s^{-1}K^{-n\_Raman}$
n_{Raman}= 5.3±0.4
R^2 = 0.9848

</td></tr>
<tr><td>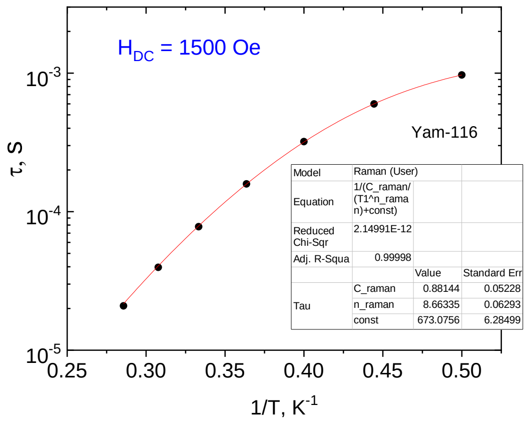
</td><td>

Raman+f(H)

$\tau^{-1} = C_{Raman} T^{n\_Raman} + f(H)$

T = 2–3.5 K
C_{Raman}= 24.9±7.5 $s^{-1}K^{-n\_Raman}$
n_{Raman}= 8.7±0.1
$f(H)$ = 673±6 s^{-1}
R^2 = 0.99998

</td></tr>
</table>

<table>
<tr>
<td>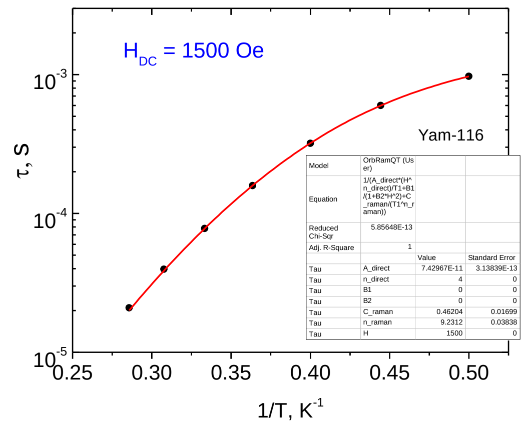
</td>
<td>Raman+Direct

$\tau^{-1} = C_{Raman}T^{n\_Raman} + A_{direct}TH^4$

T = 2–3.5 K
C_{Raman}= 0.46±0.02 $s^{-1}K^{-n\_Raman}$
n_{Raman}= 9.23±0.04
A_{direct} = $7.4{\cdot}10^{-11}{\pm}3.1{\cdot}10^{-13}$ $K^{-1}Oe^{-4}s^{-1}$
R^2 = 1</td>
</tr>
</table>

<table>
<tr>
<td>Field dependence of the relaxation time τ for complex I (H = 0.5-5 kOe, T = 2 K).</td>
<td>Fit function, temperature range, and the best-fit parameters with uncertainties.</td>
</tr>
<tr>
<td>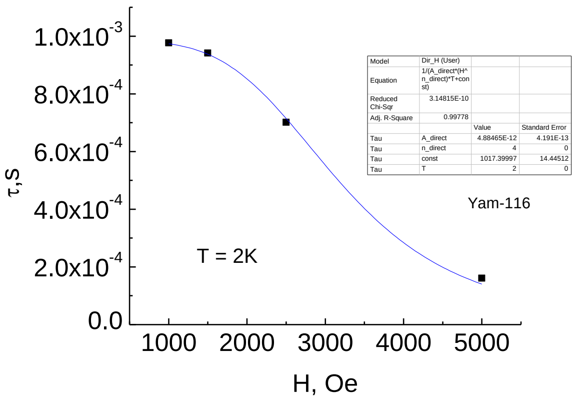
</td>
<td>Raman+Direct

$\tau^{-1} = C_{Raman}T^{n\_Raman} + A_{direct}TH^4$

T = 2 K
$C_{Raman}T^{n\_Raman}$= 1017±14 s^{-1}
A_{direct} = $4.9{\cdot}10^{-12}{\pm}4.2{\cdot}10^{-13}$ $K^{-1}Oe^{-4}s^{-1}$
R^2 = 0.9978</td>
</tr>
<tr>
<td colspan="2">As it can be seen from parameters which was obtained by the τ(1/T) dependence fit - the $C_{Raman}T^{n\_Raman}$ product should be equal to ~276 s^{-1} at 2 K. This number do not coincide with one, obtained by approximation of the field dependence τ(H). The possible reason of such mismatch can be existence of additional relaxation pathways such as quantum tunneling, which can be not negligible even in a non-zero dc-magnetic field.</td>
</tr>
</table>

<table>
<tr>
<td>Dependence of the relaxation time τ on the reciprocal temperature for II (H = 2.5 kOe, T = 2–2.5 K).</td>
<td>Fit function, temperature range, and the best-fit parameters with uncertainties.</td>
</tr>
</table>

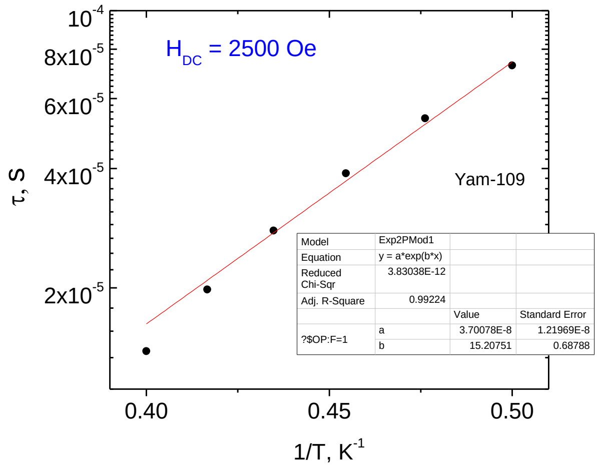

Orbach

$\tau = \tau_0 \cdot \exp\{\Delta E/kT\}$

T = 2–2.5 K

$\Delta E/k$ = 15.2±0.7 K

$\tau_0 = 3.7 \cdot 10^{-8} \pm 1.2 \cdot 10^{-8}$ s

R^2 = 0.9922

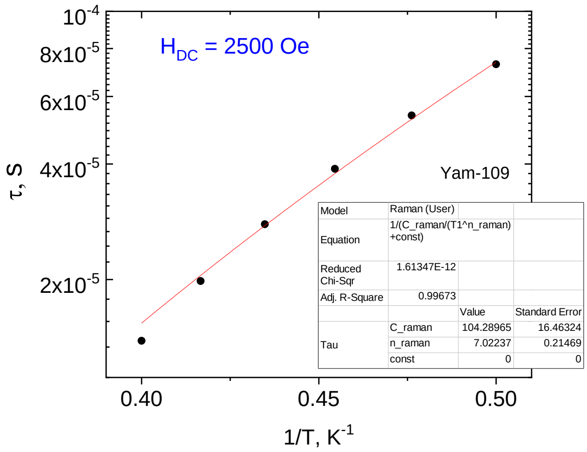

Raman

$\tau^{-1} = C_{Raman} T^{n\_Raman}$

T = 2–2.5 K

C_{Raman}= 104.3±16.5 $s^{-1}K^{-n\_Raman}$

n_{Raman}= 7.0±0.2

R^2 = 0.9967

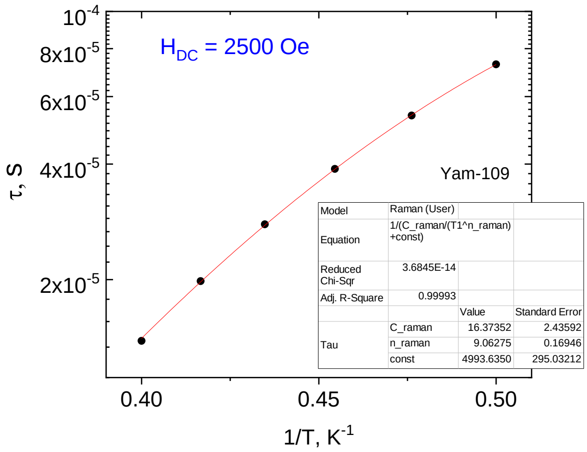

Raman+*f*(H)

$\tau^{-1} = C_{Raman} T^{n\_Raman} + f(H)$

T = 2–2.5 K

C_{Raman}= 16.4±2.4 $s^{-1}K^{-n\_Raman}$

n_{Raman}= 9.1±0.2

$f(H)$ = 4994±295 s^{-1}

R^2 = 0.99993

<table>
<tr>
<td>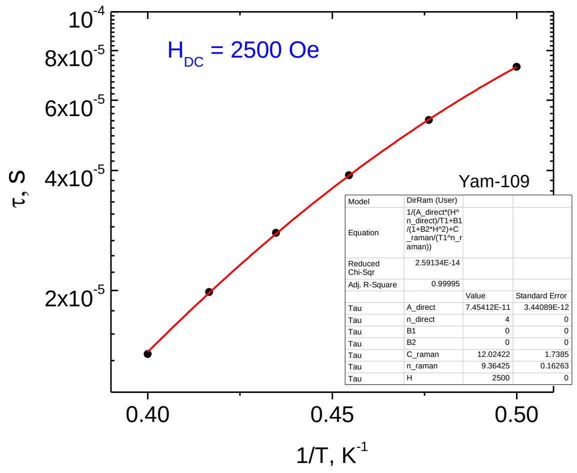
</td>
<td>Raman+Direct
$\tau^{-1} = C_{Raman}T^{n\_Raman} + A_{direct}TH^4$
T = 2–2.5 K
C_{Raman}= 12.0 ±1.7 $s^{-1}K^{-n\_Raman}$
n_{Raman}= 9.36±0.16
A_{direct} = $7.5 \cdot 10^{-11} \pm 3.4 \cdot 10^{-12}$ $K^{-1}Oe^{-4}s^{-1}$
R^2 = 0.99995</td>
</tr>
</table>

<table>
<tr>
<th>Field dependence of the relaxation time τ for complex II (H = 0.5-5 kOe, T = 2 K).</th>
<th>Fit function, temperature range, and the best-fit parameters with uncertainties.</th>
</tr>
<tr>
<td>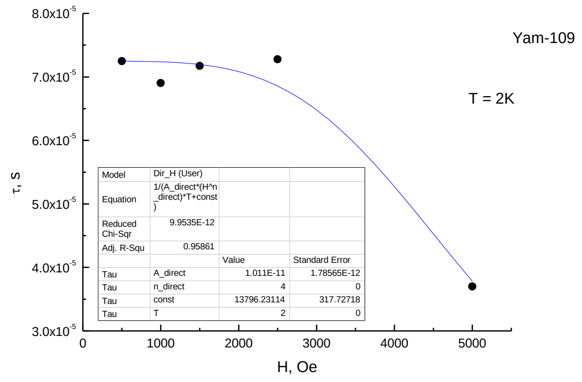
</td>
<td>Raman+Direct
$\tau^{-1} = C_{Raman}T^{n\_Raman} + A_{direct}TH^4$
T = 2 K
$C_{Raman}T^{n\_Raman}$= 13796 ±317 s^{-1}
A_{direct} = $1.0 \cdot 10^{-11} \pm 1.8 \cdot 10^{-12}$ $K^{-1}Oe^{-4}s^{-1}$
R^2 = 0.9586</td>
</tr>
<tr>
<td colspan="2">As it can be seen from parameters which was obtained by the τ(1/T) dependence fit - the $C_{Raman}T^{n\_Raman}$ product should be equal to ~7885 s^{-1} at 2 K. This number do not coincide with one, obtained by approximation of the field dependence τ(H). The possible reason of such mismatch can be existence of additional relaxation pathways such as quantum tunneling, which can be not negligible even in non-zero dc-magnetic field.</td>
</tr>
</table>
